# Analysis of Factors Affecting Individuals’ Online Consumer Credit Behavior: Evidence From China

**DOI:** 10.3389/fpsyg.2022.922571

**Published:** 2022-07-11

**Authors:** Huiying Zhao, Huaxin Peng, Wanqi Li

**Affiliations:** School of Media and Communication, Shenzhen University, Shenzhen, China

**Keywords:** online consumer credit, Triandis model, behavior, PLS analysis, China

## Abstract

In the past two decades, a growing number of Chinese young adults utilize online consumer credit to satisfy their increasing consumption demand. Although it is indeed boosting consumer markets, it has also caused many serious social problems. A number of previous studies discussed the authentication mechanism and legal supervision of online lending from a legal or economic perspective, and a small number of scholars explored factors affecting individuals’ online consumer credit from the perspective of behavioral psychology. Based on the Triandis model and existing studies on online lending, this paper constructs a theoretical model for the formation of individuals’ online consumer credit. It then adopts SmartPLS 3.00 to analyze the data set that involves 302 respondents in total. This research found that affect, facilitating conditions, perceived consequences and social factors have a significant impact on individuals’ online consumer credit. The findings of this study have political and practical implications. First, they can contribute to the rational consumption by China’s young generation and promote the sound development of online consumer credit. In the meantime, this study also helps online consumer credit platforms to provide better services and improve public relations.

## Introduction

In the past few decades, the Internet has developed into a huge global market for the exchange of goods and services (Islam, 2015). E-commerce is widely adopted all over the world. Although e-commerce in China started later than that in the United States and other developed countries due to the late development of the internet, with nearly 1 billion internet users, China has now become the world’s largest e-commerce market (McKinsey and Company, 2021b). Such tremendous change has not only promoted the logistics industry in China (Farooq et al., 2019), but also aroused a lot of water spray in the Internet financial industry. The emergence of online consumer credit provided by major e-commerce enterprises is one of them (Liu and Zhang, 2021; Zhou et al., 2021). Online consumer credit is a new kind of payment method that provides Internet-based financial services, including installment payments and micro cash loans (Han et al., 2019; Hao et al., 2019; Zhou et al., 2021). In China, lenders of online consumer credit consist of installment payment platforms represented by Fen Qi Le^1^ and Qudian^2^, as well as various financial institutions represented by commercial banks and consumer finance companies (Chen et al., 2016).

Compared with traditional consumer credit services, online consumer credit platforms are very popular amongst young people because of their convenience and lower requirements for applicants (Lee et al., 2002; Hao et al., 2019; Liu and Zhang, 2021), among which Ant Credit Pay^3^ of Alibaba and Baitiao^4^ of Jingdong Finance are typical representatives. In addition to the mainstream online shopping platforms, online takeout platforms (e.g., Meituan Takeout) and online travel booking platforms (e.g., Ctrip) also have launched their online consumer credit services. As a result, online consumer credit services have been integrated into most aspects of China’s Internet life.

However, it is worth noting that although online consumer credit represented by “e-commerce + online loans” develops rapidly and provides convenience in everyday life, it can be a two-edged sword (Feng, 2017) and we cannot overlook the risks. On the one hand, online consumer credit plays an important role in Inclusive Finance, helping to stimulate economic growth and accelerate the transformation and upgrading of the economy (Dickerson, 2008; Li and Zhu, 2018; Li et al., 2020). On the other hand, online consumer credit has also caused many social problems. According to an authoritative survey, amongst online consumer credit users in China, the post-90s and post-00s account for the highest proportion, up to 49.31%, ranking first amongst their peers in Asia (Rong 360, 2019). In recent years, with the phased expansion of the e-commerce market pioneered by companies such as Ant Credit Pay and Baitiao, the proportion of excessive and impulsive consumption by China’s Generation Z who grew up in the Internet era reached a record high level. Online consumer credit, which is fully integrated with online shopping, provides them with the capital to consume excessively (McKinsey and Company, 2021a). They are gradually developing the consumption habit of spending tomorrow’s money today.

Nevertheless, it should be noted that most young people at this age are still students or have just begun to work. Excessive consumption has brought a great burden on their normal life and work. According to Nielsen’s report (2019) on the debt situation of Chinese young people, only 42.1% of those who use credit products can pay off in the same month, which means that more than half of them are in debt because they are unable to pay off their loans in time. Overindebtedness forces a large number of people to repay loans with loans, or even turn to informal online loans such as naked loans and Taolu loans^5^ to avoid overdue payment, until eventually falling into a vicious cycle of debt (Liu and Keane, 2021). This has also led to the frequent occurrence of extreme events, and in recent years, a number of young people have been found with serious psychological problems or even have suicidal ideation when they are unable to repay their loans^6^. Apart from psychological issues, the vicious cycle of debt also, to some extent, leads to an increasing rate of bad debts in the online credit market, which is unfavorable for the sound development of the social economy.

Previous studies have discussed online consumer credit from legal, economic, and commercial perspectives, and their foci vary from the differences between traditional financial markets and online consumer credit platforms (Cortina Lorente and Schmukler, 2018; Li, 2020), supervision and regulation of online consumer credit (Liu, 2013; Rigbi, 2013; Chen, 2021; Liu and Keane, 2021; Tang and Tang, 2022), credit authentication mechanism (Wang and Liao, 2014; Chen et al., 2020), consumer credit model (Zhou and Wei, 2020) and risk assessment of online consumer credit (Chen et al., 2015; Xiao et al., 2015), to factors affecting the success of individuals’ online lending (Herzenstein et al., 2011; Duarte et al., 2012; Chen et al., 2015) and factors affecting consumers’ choice of online consumer credit (Yao et al., 2019; McDonald and Dan, 2021). However, only a limited number of studies explored factors affecting individuals’ online consumer credit behavior from the perspectives of consumers and behavioral psychology. In addition, the discussion on this issue mostly stays at the level of phenomenon analysis, especially in the Chinese context.

Thus, this study is driven by one main research question: What are the main factors affecting individuals’ online consumer credit behavior? The contributions of this study are three-fold: (1) providing a full picture to understand factors affecting the young generation’s online consumer credit based on Triandis model, (2) exploring the main factors (e.g., affect, social factors, facilitating conditions, and perceived consequences) that may have an effective impact on individuals’ online consumer credit, and (3) establishing a correct concept of consumption and promoting the positive development of online consumer credit.

## Literature Review, Research Model and Hypotheses

### Literature Review

Studies on individuals’ attitudes, perception, affect, behavioral intention, habits, social factors, and facilitating conditions often have an important impact on the occurrence of behavior (Fishbein and Ajzen, 1977; Triandis, 1980; Davis, 1989; Ajzen, 1991). Although some studies have investigated factors affecting Chinese consumers’ willingness and behavior toward online consumer credit (Sui, 2018; Hao et al., 2019; Hu and Chen, 2019; Zhu et al., 2020; Zhang et al., 2021), the focus of these studies is usually limited to college students, and they often lack a complete theoretical framework, thus they are categorized as fundamental exploratory research.

Yet the rigorous empirical analysis supported by a theoretical framework is even more scarce. So far, there are a few attempts to examine factors affecting individuals’ use of online banking and P2P lending platforms, which are very inspiring. For example, Han et al. (2019) used the Technology Acceptance Model (TAM) to analyze factors affecting Chinese consumers’ use of P2P lending platforms, proposing that financing knowledge and risk attitude are two key factors associated with P2P borrowing; Kumra et al. (2021) also used the Theory of Planned Behavior (TPB) when analyzing factors affecting the willingness of Indian BOP manufacturers to use P2P lending platforms, finding that while fast and easy access to P2P lending can favorably impact borrowers’ intention to participate, lenders are positively influenced by the high returns and diversified risk; Lee (2009) used the Technology Acceptance Model (TAM) and the Theory of Planned Behavior to analyze factors affecting the adoption of online banking in Taiwan, finding that the intention to use online banking is adversely affected mainly by the security/privacy risk and financial risk, and it is positively affected mainly by perceived benefit, usefulness, as well as attitudes; Karjaluoto et al. (2002) used the Theory of Reasoned Action (TRA) and the Technology Acceptance Model (TAM) in their research on factors affecting individuals’ use of online banking in Finland, concluding that prior personal banking experience, demographic factors (especially occupation and household income), as well as attitudes toward computers influence both attitudes and actual behavior toward online banking; Carranza et al. (2021) used the Technology Acceptance Model (TAM) to analyze factors that influence bank customers’ attitudes toward e-banking for feedbacks on banking services and the process of value co-creation, and the result showed that when e-banking users have a positive attitude toward using e-banking, they will have a greater intention to use e-banking; Ullah et al. (2022) studied the impact of Pakistani consumers’ financial skills and digital literacy on their intention to adopt m-payment/m-banking using the Technology Acceptance Model (TAM), finding that on the basis of perceived ease of use, individuals’ digital literacy can have a strong association with their intention. Amongst them, it can be observed that the TAM model is widely used in research related to the adoption of emerging technologies (Pikkarainen et al., 2004; Tan et al., 2012; Hossain et al., 2020), and it is an effective approach to achieving research goals.

This study selected Triandis model as the theoretical basis mainly because (1) in the study of individuals’ behavior, attitudes, behavior intentions, social factors usually have an important impact on the occurrence of behavior (Zhu et al., 2017). Triandis model explains the complex combination of man behaviors influenced by social, emotional, cognitive and facilitating conditions more comprehensively (Jeon et al., 2011); (2) the Triandis model has been used as a substitute for the Technology Acceptance Model (TAM), and has been applied in research on individuals’ adoption of new technologies or systems (Venkatesh et al., 2003), proving that it is suitable for explaining social behavior in new acceptance contexts (Jeon et al., 2011). Since online consumer credit is considered a new technology that can be adopted by consumers (Han et al., 2019), this paper attempts to explore the main factors affecting individuals’ online consumer credit based on the Triandis model and the existing research.

### Research Model and Hypothesis

As an important theory for the decision-making process of individuals, one of the basic assumptions of the Theory of Reasoned Action is about whether an individual adopts a certain behavior that can be controlled or determined by himself (Fishbein and Ajzen, 1975). However, in practice, individuals’ decision-making behavior is often affected by external factors. One cannot fully control their attitude and behavior (Zhu et al., 2017). Triandis (1980) proposed the Triandis model on the basis of the TRA. The Triandis model holds that there is an influence mode of “attitude-intention-behavior” in individuals’ actual behavior. To be more specific, one’s behavior is influenced by personal factors (affect and perceived consequences), social factors, and organizational factors (facilitating conditions). Personal and social factors affect individuals’ behavior by influencing one’s intentions, while organizational factors affect behavior directly (Triandis, 1980). Similar to the research of Thompson et al. (1991), in this study, we skip the mediating variable (intention) and test the direct relationship between personal factors, social factors, organizational factors and individuals’ online consumer credit, therefore paying more attention to the actual behavior involving online consumer credit.

**FIGURE 1:**
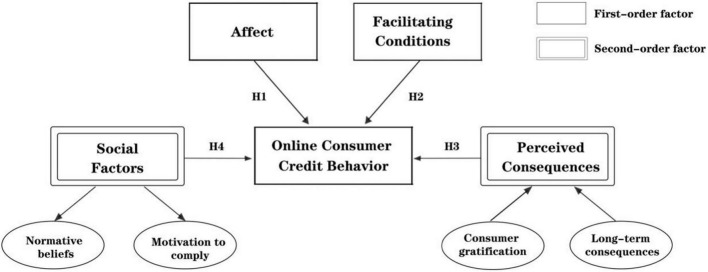
Research model.

Furthermore, this study regards the dimensions of the social factors variables (normative beliefs and motivation to comply) and the dimensions of perceived consequences variables (consumer gratification and long-term consequences) as the first-order factors, designates the social factors variables and the perceived consequences variables as the second-order factors, and puts them at the same level as affect and facilitating conditions. Because this study mainly focuses on the relationship between the second-order factors and the individuals’ online consumer credit behavior, rather than its subordinate factors (such as normal beliefs and motivation to comply). Therefore, focusing on the first-order and second-order factors can help us to simplify and highlight the main body of the study, make the variables concerned in this study more accurate at the same time. The research model is shown in Figure 1.

#### Affect

Attitude is an idea charged with affect (Triandis, 1971). Triandis (1980) defines affect as “the feelings of joy, elation, or pleasure, or depression, disgust, displeasure, or hate associated by an individual with a particular act,” and argues that precision should be achieved by separating the affective and cognitive components of attitude in attitude-behavior research. The relationship between affect and behavior has been investigated by many studies. In their study on motivation toward computer use, Davis et al. (1992) regard the enjoyment as an internal motivation of individuals’ behavior and believe that enjoyment positively affects the behavior of computer users. Karjaluoto et al. (2002) also find that individuals’ positive feelings about the use of online banking can encourage online banking behavior. Relevant studies based on the COVID-19 also found that changes in customers’ attitudes, cognition, perception and sentiments caused by the epidemic can further lead to altered purchasing decisions (Birtus and Lăzăroiu, 2021; Watson and Popescu, 2021). Therefore, we propose the following hypothesis:

**H1. Individuals’ affect is positively related to their level of online consumer credit behavior.**

#### Facilitating Conditions

Triandis (1980) defines facilitating conditions as “objective factors, “out there” in the environment, that several judges or observers can agree make an act easy to do.” He believes that if the objective conditions in the environment prevent the behavior from happening, the behavior will not happen. The positive impact of facilitating conditions on behavior has been proved by several studies. Thompson et al. (1991) propose in the research on factors affecting computer adoption that by training users and providing help when they encounter difficulties, some potential obstacles of use can be reduced or eliminated, which has a positive impact on improving the computer adoption rate. Valaskova et al. (2021) found in a study on the changes in consumers’ purchase patterns as a consequence of the COVID-19 pandemic that services will become one of the decisive factors for consumers’ purchase behavior. Musova et al. (2021) also proposed that if businesses react to consumer expectations regarding circularity by offering products and services with environmental benefits may increase their competitiveness. Furthermore, studies on online lending have also proved that convenience is the main selling point of online lending platforms. Booming online lending platforms and simple online lending operations lead to more frequent use of Fin-tech lending services, proving that objective conditions can positively affect individuals’ online lending behavior (Chaffee and Rapp, 2012; Kurniawan, 2019; Kumra et al., 2021). Therefore, we propose the following hypothesis:

**H2. Facilitating conditions are positively related to individuals’ online consumer credit behavior.**

#### Perceived Consequences

Triandis (1980) believes that, similar to the expectancy theory of motivation (Porter and Lawler, 1968), the relationship between perceived consequences and behavior can also be understood as that individuals evaluate the consequences of their behavior according to potential rewards and choose behavior according to the desirability of the rewards. Since the structure of perceived consequences is not one-dimensional, it may have several components (Triandis, 1971). Thompson et al. (1991) divide the consequences into complexity, job fit (near-term consequences) and long-term consequences in their research on the impact of computer adoption. However, according to the existing research on online consumer credit, we split perceived consequences into two dimensions: expected consumer gratification (near-term consequences) and long-term consequences. In this paper, the expected consumer gratification can be understood as immediate gratification. Some studies believe that in the process of consumption, consumers are more concerned with their current interests than with the future rewards flow (in psychological literature). Because of their preference for immediate gratification, people tend to indulge excessively in activities with immediate rewards and delayed costs (O’Donoghue and Rabin, 1999, 2000; Chang and Cheung, 2001). In the context of online consumer credit, consumers who have low spending power can turn to online consumer credit and fulfill their current consumption desire. Long-term consequences refer to the belief that a certain behavior will be rewarded in the future, such as a better chance of engaging in more meaningful work (Thompson et al., 1991). For some people, choosing online consumer credit is not so much about their current consumption desire, but about their future planning. This is especially the case for economically disadvantaged individuals who may use online consumer credit to invest in themselves and create better working and living conditions, which is more conducive to their long-term development. Thompson et al. (1991) found that people prefer to use computers when they think that using computers can provide greater help for their future work. The empirical results of Tamjidyamcholo et al. (2020) suggested that when people believe that the adoption of MOOC contributes to short-term knowledge growth and long-term social interaction, the actual utilization rate of MOOC will be greatly improved. In an explorer study on academic staff perception toward blended learning, Anthony (2021) found that long-term consequences significantly affect the lecturers’ adoption of this new teaching model. In addition, a study on the Chinese farmers’ adoption of consumer credit found that farmers’ wealth expectations have a positive impact on their consumer credit behavior (Tu, 2021). Therefore, this study puts forward the following assumption:

**H3. Individuals’ perceived consequences from online consumer credit are positively related to their level of online consumer credit behavior.**

#### Social Factors

Triandis (1971) believes that individuals’ behavior is influenced by social norms which rely on the messages received from others and reflect what individuals think they should do. In the follow-up study, Triandis (1980) regards social norms as a social factor and defines social factors as “the individual’s internalization of the reference groups’ subjective culture, and specific interpersonal agreements that the individual has made with others, in specific social situations”. The influence of social factors on behavior has been discussed in a large number of studies. Zhu et al. (2017) find that normative beliefs and motivation to comply have a positive impact on individuals’ knowledge flow behavior. Susanty et al. (2021) find that subjective norms have significant positive effects on purchase intention for green personal care products. Consumer socialization theory holds that by learning from social agents (such as parents, family members, media, peers, etc.), consumers can acquire relevant skills, knowledge and attitudes as consumers (Ward, 1974; Churchill and Moschis, 1979; Moschis and Moore, 1984). Limbu (2017) argues that this theory can be used to explain how college students learn to use credit cards or get support from their social agents. In a study on P2P lending behavior, Lee and Lee (2012) find that the decisions of others significantly affect the behavior of lenders, leading to a phenomenon called herding behavior. Serido et al. (2015) and Tang (2017) find that parents’ financial behavior affects children’s financial behavior directly, and lovers’ and friends’ financial behavior can also have an effect. Oksanen et al. (2017) also find that in a family, parents significantly affect their children’s attitude and behavior toward consumer credit. In addition, according to McDonald and Dan (2021), the change in transaction scenarios brought about by Ant Credit Pay has greatly stimulated people’s choice of online consumer credit. Therefore, we propose the following hypothesis:

**H4. Individuals’ exposure and compliance to social factors are positively related to their level of online consumer credit behavior.**

## Method

### Research Design and Data Collection

In order to test the main hypotheses of this study, we conducted a nationwide questionnaire using Wen Juanxing^7^ based on the guidelines of institutional ethical committee. Before filling out the questionnaire, all respondents have been informed of the purpose of this study as well as relevant privacy protection commitments. Since the main group of this study is individuals who have used online consumer credit, the first question of the questionnaire is a screening question. In other words, if the respondents have had online consumer credit experience, they will continue to fill in the questionnaire. Otherwise, they will stop answering the following questions and exit the questionnaire system, then the questionnaire will be submitted automatically.

The questionnaire of this research consists of two main parts. In the first part, we evaluated all variables of the model (affect, social factors, facilitating conditions, perceived consequences and online consumer credit) through 25 questions. For the measurement of variables, we mainly use the 5-point Likert scale which is a universal scale and also one of the most widely used approaches. In this research, the scale is subject to some change to match the focus of this study, and each item in the questionnaire is scored from 1 (= strongly disagree) to 5 (= strongly agree).

#### Affect

This paper mainly draws lessons from the scale of affect which is revised and compiled by Trafimow et al. (2004) and contains 4 items in total.

#### Social Factors

This scale is mainly based on the research of Chang and Cheung (2001) as well as Zhu et al. (2017), and is revised with respect to online consumer credit. In this scale, there are 3 items for each of the two aspects (i.e., normative beliefs and motivation to comply).

#### Facilitating Conditions

This scale involves 4 items that mainly draw on the research of Thompson et al. (1991) and Jeon et al. (2011).

#### Perceived Consequences

This paper measures the perceived consequences from the two dimensions: consumer gratification and long-term consequences. Consumer gratification involves 3 items which mainly draw on the research of O’Donoghue and Rabin (2000, 2001) as well as Purnawirawan et al. (2012); The long-term consequences include 4 items in total and are mainly built on the literature related to online consumer credit and the scale of the perceived consequences proposed by Thompson et al. (1991).

#### Online Consumer Credit Behavior

The existing literature mainly studies online credit behavior by adapting the scale of credit card use behavior, and this paper also adopts this method. Based on the research of Roberts and Jones (2001) and features of online consumer credit, we redesigned the scale of online consumer credit behavior which now has 4 items in total.

After processing and analyzing the data collected from the questionnaire, it is found that the overall Cronbach’s alpha coefficient of the scale is greater than 0.7, and the Cronbach’s alpha coefficient corresponding to the five dimensions is greater than 0.7, indicating that the internal consistency of the questionnaire is good, so the reliability of the results of this survey is excellent (see Table 1).

**TABLE 1:** Questionnaire items.

Construct		Items	Data of each dimension	Overall data
Affect		Using Ant Credit Pay/Baitiao makes the consumption process more interesting.	Alpha = 0.871	
		Using Ant Credit Pay/Baitiao makes consumption faster and more convenient.	Mean = 3.376	
		Using Ant Credit Pay/Baitiao makes the consumption forms more diverse.	S.D. = 1.023	
		Using Ant Credit Pay/Baitiao to consume makes me feel happy.		
Social factors	Normative beliefs	Their advertising is very attractive.	Alpha = 0.861	Alpha = 0.847
		My friends around me are using it and think I can use it too.	Mean = 3.364	Mean = 3.338
		My close family members are using it and think I can use it too.	S.D. = 1.111	S.D. = 0.936
	Motivation to comply	Usually, I tend to believe what they advertise.	Alpha = 0.865	
		Usually, I will be influenced by my friends and choose to take their advice.	Mean = 3.412	
		Usually, I will be influenced by my family and choose to take their advice.	S.D. = 1.089	
Perceived consequences	Consumer gratification	Using Ant Credit Pay/Baitiao can help me buy a commodity that I can’t afford at this stage.	Alpha = 0.871	Alpha = 0.855
		Using Ant Credit Pay/Baitiao will meet my consumption desire in the short term.	Mean = 3.257	Mean = 3.272
		Using Ant Credit Pay/Baitiao can increase my disposable balance to some extent in order to provide financial support for other consumption behavior.	S.D. = 1.042	S.D. = 0.922
	Long-term consequences	Using Ant Credit Pay/Baitiao will improve my consumption level.	Alpha = 0.898	
		Using Ant Credit Pay/Baitiao will improve my quality of life.	Mean = 3.282	
		Using Ant Credit Pay/Baitiao can relieve my work pressure to some extent.	S.D. = 1.131	
		Using Ant Credit Pay/Baitiao can reduce my burden of life to some extent.		
Facilitating conditions		Now major consumer platforms are competing to launch products of consumer credit, and there are a variety of platforms for me to choose from.	Alpha = 0.907	
		The major platforms of online consumer credit provide a variety of installment options (such as 3 months, 6 months or 12 months, etc.).	Mean = 3.315	
		The operation of online consumer credit is simple, and I can easily master and use it.	S.D. = 1.145	
		The customer service of major online consumer credit platforms is enthusiastic and they can solve my problems in time.		
Online consumer credit behavior		I would like to know all kinds of information about online consumer credit products (such as Ant Credit Pay and Baitiao, etc.).	Alpha = 0.869	
		I am willing to use online consumer credit such as Ant Credit Pay/Baitiao.	Mean = 3.479	
		I often use online consumer credit such as Ant Credit Pay/Baitiao.	S.D. = 0.945	
		I am willing to try more types of online consumer credit products.		

*In order to facilitate the understanding of the respondents, all items in the questionnaire adopt the expression Ant Credit Pay/Baitiao instead of online consumer credit.*

The second part surveys the basic situation of the respondents. Table 2 lists the characteristics of all respondents. We used snowball and convenient sampling approaches to recruit samples, and all of the respondents filled out the questionnaire online. Each respondent received 3 yuan (CNY) as a questionnaire reward so as to encourage them to answer carefully. Moreover, in order to ensure the accuracy of the questionnaire and minimize the sampling deviation caused by non-random sampling, we set interference items in the questionnaire and stipulated the minimum and maximum answering time. From January 15, 2022, to February 21, 2022, 362 questionnaires were collected. After screening and eliminating 13 invalid questionnaires, 349 valid questionnaires were obtained, with an effective rate of 96.4%. The valid questionnaires were then further screened based on the question of whether the respondent has ever used online consumer credit, and 47 of them were screened out because these respondents did not have such experience. Therefore, a total of 302 respondents’ data were used for the final analysis.

**TABLE 2:** Respondent profile.

Measure	Category	Frequency	Percent (%)
Gender	Male	108	35.76
	Female	194	64.24
Age	18-26	287	95.03
	27-36	11	3.64
	37-46	4	1.32
	Above 46	0	0
Occupation	Student (such as junior high school student/college student, etc.)	160	52.98
	Service staff (catering waiter/driver/salesman, etc.)	35	11.59
	Office worker	71	23.51
	Civil servant/Government staff	25	8.28
	Unemployed	9	2.98
	Others	2	0.66
Source of income	Living expenses provided by the family	133	44.04
	Part-time job	51	16.89
	Subsidy by the government/school	16	5.30
	Wages	98	32.45
	Loan	2	0.66
	Others	2	0.66
The experience of supporting loans with loans	Yes	181	59.93
	No	121	40.07

Furthermore, due to the existence of second-order factors in this study, PLS method can form potential structure from reflective indicators and formative indicators, respectively (Chin, 1988), and can also construct a hybrid model with both reflective and formative structures. Therefore, in this study, we used SmartPLS 3.0 for data analysis and processing.

### Data Analysis

#### Descriptive Statistics

Descriptive analysis reveals that there are significant gender differences in online consumer credit. Females account for 64.24% of the total users. This is consistent with the findings of Lyons (2004) and Robb (2011), who found that females are more likely to engage in credit behavior, which may be related to the fact that the consumption demand of women is greater than that of men in the consumer market (Fischer and Arnold, 1990; Shkurkin et al., 2017). As for the occupation of participants, most of them are students (such as junior/high school students/college students), representing 52.98% of the total. In terms of age distribution, respondents are mainly young people aged 18-26, accounting for 95.03%. These findings are consistent with the view of Lee et al. (2002), who state that age is related to the use of online lending platforms, because young people are usually more inclined to use them. From the perspective of income sources, the main source of income of most respondents is the living expenses provided by their families, and this is the case for a total of 133 people, accounting for 44.04%. And more than half of them (59.93%) have had the experience of supporting loans with loans. The data further affirms the importance and urgency of our research. A thorough understanding of the main factors that affect young people’s online consumer credit is the premise of finding specific ways to provide timely intervention and facilitate rational usage of online consumer credit products.

#### Measurement Model

Before examining the structural relationships, we conducted a confirmatory factor analysis to evaluate the measurement model (see Table 3). Since this research model contains two second-order variables (social factors and perceived consequences), we use the factor scores of the first-order constructs to create superordinate second-order constructs. We regard the sub-dimensions of social factors as reflective indicators and the sub-dimensions of perceived consequences as formative indicators. In order to verify the measurement model, we evaluated the convergent validity and the discriminant validity. In this paper, the composite reliability (CR) and the average variance extracted (AVE) are used as the evaluation criteria for the convergent validity. When the CR value of each factor is greater than 0.7 and the AVE value is greater than 0.5, it is generally considered that the convergent validity is acceptable (Fornell and Larcker, 1981; Hair et al., 1998). In this study, composite reliabilities ranged from 0.892 to 0.935, and the average variance extracted was 0.717-0.787, indicating that the convergence validity of this dimension is satisfactory. Furthermore, when one factor’s square root value of AVE is larger than the correlation coefficient between this factor and other factors, it indicates that the discriminant validity is high. It can be seen from Table 3 that for each factor, the square root value of AVE is greater than any correlation coefficient, which proves the discriminant validity of the study. We used the structural equation model (SEM) to analyze the data. Table 4 summarized the correlation matrix of all variables, and there is no multicollinearity threat between structures since every variance inflation factor (VIF) values ranged from 1.000 to 1.518.

**TABLE 3:** Results of PLS confirmatory factor analysis.

Measure		Items	CR/AVE	Loading	Standard error	t-value
Affect		Using Ant Credit Pay/Baitiao makes the consumption process more interesting.	0.911/0.72	0.828	0.020	41.705
		Using Ant Credit Pay/Baitiao makes consumption faster and more convenient.		0.870	0.014	64.121
		Using Ant Credit Pay/Baitiao makes the consumption forms more diverse.		0.848	0.019	44.362
		Using Ant Credit Pay/Baitiao to consume makes me feel happy.		0.846	0.018	45.755
Social factors	Normative beliefs	Their advertising is very attractive.	0.915/0.782	0.881	0.012	75.852
(AVE = 0.726,CR = 0.841)		My friends around me are using it and think I can use it too.		0.897	0.012	77.594
		My close family members are using it and think I can use it too.		0.876	0.013	66.313
	Motivation to comply	Usually, I tend to believe what they advertise.	0.917/0.787	0.872	0.013	66.280
		Usually, I will be influenced by my friends and choose to take their advice.		0.895	0.011	81.823
		Usually, I will be influenced by my family and choose to take their advice.		0.895	0.011	83.878
Perceived consequences	Consumer gratification	Using Ant Credit Pay/Baitiao can help me buy a commodity that I can’t afford at this stage.	0.892/0.734	0.876	0.014	62.338
		Using Ant Credit Pay/Baitiao will meet my consumption desire in the short term.		0.846	0.016	52.063
		Using Ant Credit Pay/Baitiao can increase my disposable balance to some extent in order to provide financial support for other consumption behavior.		0.847	0.016	53.639
	Long-term consequences	Using Ant Credit Pay/Baitiao will improve my consumption level.	0.929/0.765	0.850	0.014	61.083
		Using Ant Credit Pay/Baitiao will improve my quality of life.		0.909	0.009	98.672
		Using Ant Credit Pay/Baitiao can relieve my work pressure to some extent.		0.869	0.013	66.252
		Using Ant Credit Pay/Baitiao can reduce my burden of life to some extent.		0.870	0.013	69.374
Facilitating conditions		Now major consumer platforms are competing to launch products of consumer credit, and there are a variety of platforms for me to choose from.	0.935/0.782	0.876	0.013	67.424
		The major platforms of online consumer credit provide a variety of installment options (such as 3 months, 6 months or 12 months, etc.).		0.878	0.012	70.555
		The operation of online consumer credit is simple, and I can easily master and use it.		0.916	0.009	97.318
		The customer service of major online consumer credit platforms is enthusiastic and they can solve my problems in time.		0.867	0.014	62.276
Online consumer credit behavior		I would like to know all kinds of information about online consumer credit products (such as Ant Credit Pay and Baitiao, etc.).	0.910/0.717	0.836	0.020	42.768
		I am willing to use online consumer credit such as Ant Credit Pay/Baitiao.		0.859	0.019	46.080
		I often use online consumer credit such as Ant Credit Pay/Baitiao.		0.854	0.015	56.860
		I am willing to try more types of online consumer credit products for consumption.		0.837	0.018	46.007

**TABLE 4:** Means, standard deviations, and correlations among key variables.

	Mean	Std. Deviation	AF	FC	OCC	SF	PC
AF	3.376	1.023	1				
FC	3.315	1.145	0.421**	1			
OCC	3.479	0.945	0.471**	0.482**	1		
SF	3.388	0.936	0.482**	0.466**	0.520**	1	
PC	3.272	0.922	0.444**	0.471**	0.503**	0.439**	1

*p < 0.05; **p < 0.01.

*AF, affect; FC, facilitating conditions; OCC, online consumer credit; SF, social factors; PC, perceived consequences.*

## Results

**FIGURE 2:**
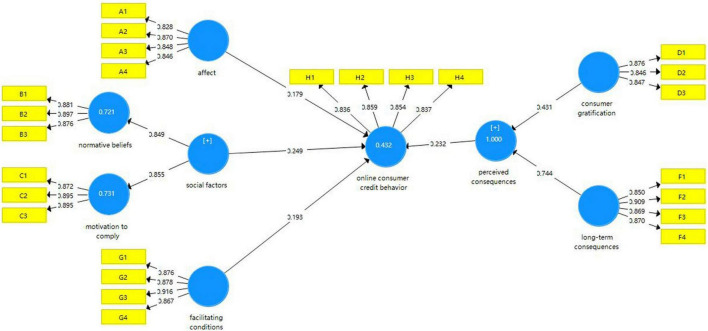
Results of PLS analysis.

Figure 2 shows the results of the structural analysis. Table 5 shows the path coefficients in the model. From the path analysis of the model, the test results support all the hypotheses of this study. Affect, facilitating conditions, perceived consequences and social factors all have a positive impact on individuals’ online consumer credit, which account for about 42% variance of such behavior.

**TABLE 5:** Path analysis.

Path	Variable relation	Path coefficient	Std. deviation	T-statistic	*P*-value
Path 1	affect - > online consumer credit	0.179	0.050	3.559	0.000
Path 2	facilitating conditions - > online consumer credit	0.193	0.050	3.836	0.000
Path 3	perceived consequences - > online consumer credit	0.232	0.056	4.166	0.000
Path 4	social factors - > online consumer credit	0.249	0.055	4.546	0.000

Firstly, affect has a significant positive impact on online consumer credit (β = 0.179, *P* < 0.05), supporting hypothesis 1. When one holds a more positive view of online consumer credit, he/she is more likely to use it, which proves that online consumer credit is affected by individuals’ intrinsic motivation. This is consistent with the research findings of Norvilitis et al. (2006). They state that young adults who lack financial knowledge are more likely to be overburdened with debt. Therefore, at this point, we believe it is very important to guide individuals to establish a correct understanding of online consumer credit. In other words, it is necessary for them to have a certain level knowledge about credit finance.

Meanwhile, this study found that facilitating conditions have a significant positive impact on online consumer credit (β = 0.193, *P* < 0.05), supporting hypothesis 2. As an objective factor in the environment, facilitating conditions are the prerequisite for behavior (Triandis, 1980). Without the blowout in online consumer credit platforms and their service support, the market of online consumer credit will be far from its current scale. However, with the further expansion of China’s online consumer credit market, many illegal online consumer credit platforms such as Taolu loans and naked loans have been increasing as well. Generally, such platforms have been professionally packaged and their service personnel also have been professionally trained. Under the guise of fast approval and low threshold of loans, they use a set of mature fraud systems to induce individuals to borrow (Liu and Keane, 2021). Therefore, relevant authorities should strengthen the supervision of online credit markets, resolutely ban illegal online consumer credit platforms, and prevent young adults from entering by mistake and eventually falling into the trap of Taolu loans.

Perceived consequences have a significant positive impact on online consumer credit (β = 0.232, *P* < 0.05), supporting hypothesis 3. The perceived consequences are individuals’ outlook on the future (Thompson et al., 1991). Online consumer credit can meet the consumption desire of individuals when they do not have the consumption ability, but consumer credit itself is overdrawing the money of the future. For people who have high levels of self-control, online consumer credit can alleviate the pressure of their work and life to a certain extent. On the other hand, for people lacking self-control, online consumer credit has the potential to trigger greater consumption desire, which eventually makes their consumption far exceed their income and results in excessive debt. The huge repayment pressure can also affect their physical and mental health, causing problems such as depression, nervousness, and anger. Therefore, our society should guide young adults to establish a correct concept of consumption by providing education on the financial understanding of consumer credit, so as to help them avoid excessive consumption and achieve moderate consumption within income.

Finally, social factors have a significant positive impact on online consumer credit (β = 0.249, *P* < 0.05), supporting hypothesis 4. This result suggest that compared with the intrinsic motivational factors, the social factors are crucial to individuals’ online consumer credit behavior. When supported by important others, it will be easier for them to adopt online consumer credit. Therefore, society, schools and families should actively participate in the construction of relevant education mechanisms to help the younger generation establish a correct consumption concept, cultivate certain financial credit knowledge, and learn to control their online consumer credit desire.

## Discussion

In terms of the practical implications of this research, due to the complexity of China’s online credit markets, there is an urgent need for the cooperation of all sectors of society toward further standardization and management. Only when we have an in-depth understanding of the main factors affecting individuals’ online consumer credit can we find specific ways to provide timely intervention. However, technology by itself is innocent, and people are the ones who exploit its potential (Arnold and Pearce, 2015). Some studies have pointed out that online lending promotes the transformation and development of the consumer market to a certain extent (Dickerson, 2008). Considering fierce competition in the online lending market, attracting more consumers is of vital importance for each platform to stand out from their competitors. The results of this study not only provide a reference for standardizing the online consumer credit market, but also help some online consumer credit platforms to achieve better services and public relations.

Specifically, our results indicate that individuals’ affect is positively related to their level of online consumer credit behavior. This is consistent with the research findings of Gärling et al. (2020). They state that individuals’ negative attitude toward borrowing can decrease their choices of months of installment payments. Accordingly, the more positive an individual’s attitude toward online consumer credit, the more likely he/she is to take action. Therefore, Online consumer credit platforms can promote individuals’ positive emotions by carrying out different activities, and they can also attract public attention through novel forms of advertising.

The impact of facilitating conditions on individuals’ online consumer credit behavior is significant and consistent with the previous research (Chen et al., 2021). Individuals’ satisfaction to Internet consumer finance platforms has a larger positive effect on their continuous use intention. Therefore, Online consumer credit platforms should weigh heavier on technology innovation and provide better services to gain and maintain customers as well.

The impact of perceived consequences on individuals’ online consumer credit behavior is also consistent with previous findings (Chen et al., 2021; Liu and Zhang, 2021). Customers’ perception of online consumer credit behavior is affected by the credit limit. Higher credit limit is easier to stimulate the impulse buying desire of the customers (Pradhan et al., 2018). However, if the financial literacy of customers is low, it might cause them to bear huge repayment pressure. In the meantime, the recovering funds cost of online consumer credit platforms will be higher, and the probability of bad debts will be greater. Therefore, the online consumer credit platforms should establish a scientific and reasonable evaluation mechanism to ensure the credit limit to be allocated according to different consumption capacities of customers. In addition, to achieve the purpose of attracting customers with a high standard credit system on the basis of curbing their impulse buying behavior, the platforms should strictly abide by the basic interest rate set by the state.

Lastly, our results indicate that individuals’ online consumer credit is most affected by social factors, which also consistent with previous findings (Gao and Wang, 2020). They state that in terms of online consumer credit, individuals’ behavior is significantly affected by group behavior (of families, friends, etc.), which means that there is an obvious peer effect. Furthermore, the research of Hao et al. (2019) found that if the media or campus advertisements publicize more negative information about online loans, it will regulate the demand of young adults for online consumer credit. In other words, advertising also plays an important role in individuals’ decision about whether or not to use online consumer credit. Therefore, implementing appropriate publicity and enhancing platform reputation can help platforms to gain and maintain customers as well.

## Conclusion

Based on the Triandis model and relevant literature, this research constructed a theoretical model affecting individuals’ online consumer credit behavior. Using this model, we identified and validated the main factors affecting individuals’ online consumer credit behavior. According to the literature on technology adoption and online consumer credit, the social factors were further divided into normative beliefs and motivation to comply. The perceived consequences were subdivided into two dimensions: consumer gratification and long-term consequences. The results confirm that affect, facilitating conditions, perceived consequences and social factors all significantly affect individuals’ online consumer credit, amongst which social factors have the greatest impact. According to the results of this study, we provide feedbacks for online lending platforms to achieve better services and public relations. On top of that, we also put forward several suggestions for the industry and our society, such as all sectors of our society (especially relevant government departments) should guide individuals to establish a correct understanding of online consumer credit; the education sector should provide education on the financial understanding of consumer credit; supervisory agencies need to strengthen online credit market supervision and resolutely ban illegal online consumer credit platforms; and online consumer credit platforms should carry out effective media campaigns. Their joint effort will contribute to the long-term development of the online consumer credit industry and the wellbeing of the customers.

This study has certain limitations. Firstly, we mainly focus on the direct impact of affect, facilitating conditions, perceived consequences and social factors on online consumer credit. We do not verify the impact of intermediary variables or adjustment variables on the relationship between independent variables and dependent variables. Therefore, future studies may add intermediary variables or adjustment variables to enrich the research model; Secondly, the sample size of the study is limited. Compared with a sample of 300 respondents, we hope that future research can expand the sample size so as to further enhance the reliability of the research results. In addition, while carrying out preliminary interviews and reviewing the literature, we found that (1) different consumption patterns of online lending will affect the consumption choices of young adults. They are more inclined to choose the online consumption mode provided by the same payment platform, for example, using Ant Credit Pay while shopping online on Taobao. However, when individuals face different consumption situations, such as loaning to personal account from an online lending platform and then consume on other platforms, they will hesitate. (2) Gender discrimination against women in the online lending market exists not only in China, but also all over the world. However, scholars around the world pay little attention to this issue. Therefore, we look forward to the supplement of follow-up research.

## Data Availability Statement

The raw data supporting the conclusions of this article will be made available by the authors, without undue reservation.

## Ethics Statement

The studies involving human participants were reviewed and approved by Shenzhen University Ethics Committee. The participants provided their written informed consent to participate in this study. Written informed consent was obtained from the individual(s) for the publication of any potentially identifiable images or data included in this article.

## Author Contributions

HZ created the main framework of this study and made appropriate modifications under the guidance of HP. WL provided help to collect data, wrote some results, and participated in the discussion. HZ wrote and completed this article. HP guided the revision of the study in the whole process. All authors contributed to this article and approved the submitted version.

## Conflict of Interest

The authors declare that the research was conducted in the absence of any commercial or financial relationships that could be construed as a potential conflict of interest.

## Publisher’s Note

All claims expressed in this article are solely those of the authors and do not necessarily represent those of their affiliated organizations, or those of the publisher, the editors and the reviewers. Any product that may be evaluated in this article, or claim that may be made by its manufacturer, is not guaranteed or endorsed by the publisher.

## Footnotes

1 As an installment shopping APP, Fen Qi Le was launched in October 2013. It is the pioneer of China’s installment shopping e-commerce model. In addition to providing installment shopping services, Fen Qi Le also provides small loans services for users over the age of 18.

2 Similar to Fen Qi Le, Qudian is a platform for installment shopping and provides small loans services at the same time.

3 Ant Credit Pay is an online consumer credit product launched by Alibaba in April 2015. According to their credit degree, consumers will get a consumption credit line ranging from 500 yuan to 50,000 yuan.

4 Baitiao is the first online consumer credit product in China, launched by Jingdong finance in February 2014.

5 According to the interpretation of the Ministry of public security of China, Taolu loans is a general description of the relevant illegal and criminal activities which induce or force the victim to sign relevant agreements such as loan or disguised loan, mortgage and guarantee for the purpose of illegal possession in the name of private lending, and form false claims and debts by falsely increasing the loan amount, maliciously creating a breach of contract, arbitrarily identifying a breach of contract, destroying and concealing repayment evidence, and by means of litigation, arbitration, notarization or violence.

6 It is reported that Xu, a college student who just graduated from a university in Jiangsu in 2020, failed to repay dozens of online loans in time and then suffered from depression. In the end, he could no longer bear the pressure and jumped off from a hotel and committed suicide.https://baijiahao.baidu.com/s?id=1662053521625104631andwfr=spiderandfor=pc.

7 An online questionnaire survey platform in China, similar to SurveyMonkey.
